# Mapping trust relationships in organ donation and transplantation: a conceptual model

**DOI:** 10.1186/s12910-023-00965-2

**Published:** 2023-11-01

**Authors:** María Victoria Martínez-López, Leah McLaughlin, Alberto Molina-Pérez, Krzysztof Pabisiak, Nadia Primc, Gurch Randhawa, David Rodríguez-Arias, Jorge Suárez, Sabine Wöhlke, Janet Delgado

**Affiliations:** 1 Department of Philosophy I, FiloLab-UGR, University of Granada, Granada, Spain; 2 Ethical Legal and Psychosocial Aspects of Organ Transplantation (ELPAT), European Society for Organ Transplantation (ESOT), Padua, Italy; 3 School of Medical and Health Sciences, Bangor University, Bangor, UK; 4 Instituto de Estudios Sociales Avanzados (IESA), CSIC, Córdoba, Spain; 5 Dept Nephrology Transplantation and Internal Medicine, Pomeranian Medical University, Szczecin, Poland; 6 Institute of History and Ethics of Medicine, Medical Department, Heidelberg University, Heidelberg, Germany; 7 Institute for Health Research, University of Bedfordshire, Bedfordshire, UK; 8 Department Health Sciences, Faculty Life Science, Hamburg University of Applied Sciences, Hamburg, Germany

**Keywords:** Trust, Organ donation, Organ transplantation, Conceptual model, Trust networks, Clinical relationship

## Abstract

The organ donation and transplantation (ODT) system heavily relies on the willingness of individuals to donate their organs. While it is widely believed that public trust plays a crucial role in shaping donation rates, the empirical support for this assumption remains limited. In order to bridge this knowledge gap, this article takes a foundational approach by elucidating the concept of trust within the context of ODT. By examining the stakeholders involved, identifying influential factors, and mapping the intricate trust relationships among trustors, trustees, and objects of trust, we aim to provide a comprehensive understanding of trust dynamics in ODT. We employ maps and graphs to illustrate the functioning of these trust relationships, enabling a visual representation of the complex interactions within the ODT system. Through this conceptual groundwork, we pave the way for future empirical research to investigate the link between trust and organ donation rates, informed by a clarified understanding of trust in ODT. This study can also provide valuable insights to inform interventions and policies aimed at enhancing organ donation rates.

## Introduction

Trust plays a pivotal role in medical contexts, including transplantation medicine, which relies on a complex network of trust and mistrust relationships [1]. The success of organ donation and transplantation (ODT) is a collaborative achievement involving policymakers, institutions, healthcare professionals, donors, recipients, and families, who foster cooperation and mutual trust [2]. Various factors influence individuals and families willingness to donate organs, including consent policies^1^, perceptions of the body, understanding of death, engagement in end-of-life rituals, as well as altruistic and solidarity values [3–7].

Although it is widely believed that increased trust can enhance the willingness to donate [8–11], empirical evidence supporting a causal relationship between trust and increased donation (or willingness to donate) is limited.

Conversely, greater public distrust is generally associated with reduced organ donations [12, 13]. Consequently, measures have been taken to address specific donation-related scandals, such as the establishment of agencies aimed at restoring public trust [14], the development or revision of laws and policies, and substantial investments in positive publicity campaigns [1]. While building trust in transplantation medicine can be accomplished through appropriate practices and regulatory frameworks [15], there are other influencing factors that can affect trust, such as the body concept, the underlying idea of autonomy, or cultural aspects related to solidarity or reciprocity, among others. It is crucial to recognise that cultivating greater trust in healthcare systems is central to the ethical aspects of (ODT), rather than being ancillary or irrelevant [2]. For this purpose, it is necessary to clarify what are the main aspects that constitute and affect trust in ODT.

This research aims to comprehend trust relationships in ODT from the perspective of stakeholders. We propose a comprehensive model that elucidates the key dimensions of trust and its various components by identifying trustors, trustees, and objects of trust. The main research question of this article is: What constitutes trust and how is it distributed among the actors involved in the ODT process? To address this question, we present a conceptual model that serves as a foundational framework for future empirical research, including the development of scales or questionnaires to identify and measure trust and mistrust relationships among the diverse actors engaged in ODT. Moreover, this model can provide a basis for exploring the causal relationship between trust and willingness to donate, shedding light on the factors influencing individuals’ decisions regarding organ donation.

## Conceptual clarifications

### ‘Trust’, ‘lack of trust’ and ‘mistrust’

Trust is an umbrella term that can be characterised as the belief that others (i.e. individuals, institutions) will behave as expected, which generates a positive emotion linked to a sense of security. The concept of trust is multifaceted [16] and can be approached from divergent perspectives [1]. From the perspective ofpsychology and political science, trust is conceived as a psychological event or a mental state of isolated individuals, that can be reduced to its cognitive content or to its behavioural expressions. In contrast, from a sociological perspective, trust is a property of collective units that is applicable to the relations among people rather than to their psychological states taken individually [17].

For the purposes of this article, we will assume, following [18], that trust is an essentialcondition and facilitator of social interaction and, in particular, cooperation. In this sense, Schilke defines it as “the willingness of an entity (i.e. the trustor) to become vulnerable to another entity (i.e. the trustee) taking. In this risk, the trustor presumes that the trustee will act in a way that is conducive to the trustor’s welfare despite the trustee’s actions being outside the trustor’s control” [19]. In the context of ODT, when the trustor is a deceased donor or their family, we should consider that it is not necessarily the trustor’s welfare that is at stake, but the trustor’s interests and values.

Trust has an important moral dimension because it involves an asymmetrical bilateral relationship of power and vulnerability between the trustor and the trustee. On the one hand, the trustor places theirself in a position of vulnerability to the discretionary power of the trustee, while on the other hand the trustee is morally bound by the trustor’s expectations to use their power responsibly [1].

We will assume that trustors are individuals or groups of people (e.g. potential individual donors, families, recipients, health professionals), whereas trustees can be either individuals, groups, or abstract entities such as institutions and organisations (e.g. the transplant system, policy makers). Trust can vary in intensity and nature depending on the relationship between the trustor and the trustee: it is not the same to trust a particular person, a professional role (e.g. nurse or doctor), or an abstract system [20]. However, for the sake of clarity and simplicity, we will not explore this further within this article.

Trust can be withheld without implying distrust or mistrust^2^. One may withhold trust when the conditions for rational trust are unclear for a given person or situation [2].

Lack of trust is just the absence of trust, and trust can be absent without implying distrust. This may happen when one is indifferent or disengaged about a certain issue [1, 21]. However, as Griffith notes [22], “lack of trust is often framed as something that needs to be changed in individuals who do not trust rather than something that needs to change in providers and organisations that have not demonstrated that they are trustworthy”.

Unlike the absence of trust, distrust is a logical response based on scepticism, suspicions, and concerns. In healthcare, distrust can stem from the assumptions that providers or institutions may offer unequal or variable quality of care, and that the patient may receive substandard treatment [22]. In ODT, previous research has examined mistrust in relation to cultural, system, and medical factors [23, 24]. A common source of mistrust is the perception that doctors may not try to save a potential donor’s life or may declare them dead prematurely to obtain their organs [23, 25, 26]. This issue is especially relevant among minority communities - for example in the UK [7, 27–29]. However, these studies focus on mistrust in access to the transplant system and not the health system in general.

### The elements of trust: ‘trustor’, ‘trustee’, and ‘objects of trust’

**Fig. 1:**
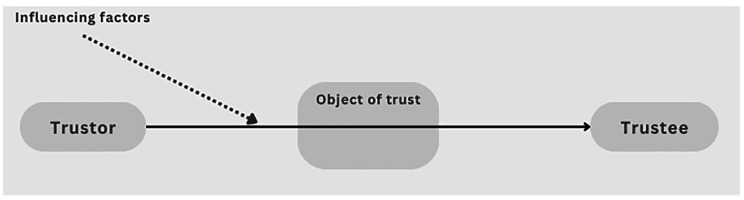
The essential elements of a trust relationship

We propose that trust relationships have three essential elements: a trustor, an object (or content), and a trustee (Fig. 1). The trustor holds (and acts or behaves based on) a belief that something is or will be [true, accurate, fair, appropriate, useful, etc.], which relies wholly or partly on the trustee. The same object of trust may involve and be attributed to multiple trustees. For instance, respecting the deceased’s decision to donate (opt-in) or not donate (opt-out) organs may depend on the doctors, the family, and the government (through policies regulating individual consent and the family’s role in the decision). Moreover, a single trustee may be responsible for (i.e. be expected to deliver) multiple trust objects. For example, people may trust or distrust doctors to save their lives, respect their dignity and their bodies after death, and honour their wishes about organ donation.

However, trust involves not only the trustor and trustee (the person, group, or entity that is being trusted), but also the object of trust (the action or outcome that is expected or desired from the trustee). The object of trust varies depending on the context and the needs or interests of the trustor (the person, group, or entity that is placing trust). For instance, the trustor may trust the family for emotional support, but not for medical guidance. Or the trustor may trust the organ transplantation system for fair allocation of organs, but not for guaranteed success of the transplant.

Trust relationships are not limited to dyadic interactions but often form complex networks that include multiple trustors, trustees, and objects of trust. In such a network, the same social actor can assume different roles as a trustor or a trustee, depending on their relationships with other social actors.

The ODT system is a interconnected matrix structure with many social players interacting with one another. This include: organ donors and their families, patients who need an organ, health professionals who care for patients and perform organ removal and transplantation, donation and transplantation system (DTS), i.e.an abstract entity that regulates and coordinates organ procurement and allocation–, legislators who set the legal and ethical framework for the DTS, and society as a whole which isthe source and destination of donated organs. In turn, these stakeholders are part of a wider system, the health system, that covers all aspects of health care in a country or region.

In this article, we focus on the allocation of trust from the trustors to the trustees. However, the reverse perspective, from the trustees to the trustors, is also relevant to understanding how trustworthiness is established or maintained, and how it facilitates ODT. Yet, relationships of trust and trustworthiness are not necessarily symmetrical and can form different networks. Therefore, from the perspective of policymakers and institutional actors, who are the ultimate trustees, it may be useful to use a different approach.

### Influencing factors

Trust relations, like almost any other socially determined relation, are hugely determined by personal views, values, and beliefs. Many features of the agents involved shape what is important to them, whom they trust for what, and the intensity and relevance of that relation. Although these features can be determinant and constituent, our model suggests treating them as influencing factors that exert an influence on these relationships. As it will be explained further below, our proposed model suggests that certain trust relationships are inherent to trust in ODT, and these influencing factors need to be considered as variables that can have extremely diverse effects and create a myriad of potential dynamic relationships.

In order to clarify how influencing factors can change the structure of a relationship, we can take the example of the trustor’s past experiences with health professionals. If somebody has had good or bad experiences in the past with a health professional, that can deeply affect their expectation on that professional’s future behaviour. That does not mean that the patient either will or will not trust the health professional to provide them with good care, but the relationship will probably be much stronger or weaker depending on this mediation variable. In this fashion, many other factors such as personal and religious beliefs, information spread by media, knowledge of the transplant system, and so on, have a strong role in changing the intensity and even determining the creation or destruction of trust relationships.

These factors are deeply contextual, but real trust relationships are rarely (if ever) fully independent of the reality in which the actors involved live. Analysing how trust relationships are affected by these influencing factors will help us show that even after conceptualising it, trust creates dynamic relationships that are deeply mediated and that can radically differ from their original status given different contexts. Thus, the mapping of trust relationships we offer up next should be understood as a model of common structures and it needs to be confronted with real scenarios to get an actual representation: cultural backgrounds, religious beliefs, personal values, public information, or past experiences are some of the many potential influencing factors that can have a great impact on the final outcome of trust relationships in real life. We will provide an example of this in Sect. 4.

## Trust Dynamics in ODT

### Mapping trust relationships

**Fig. 2:**
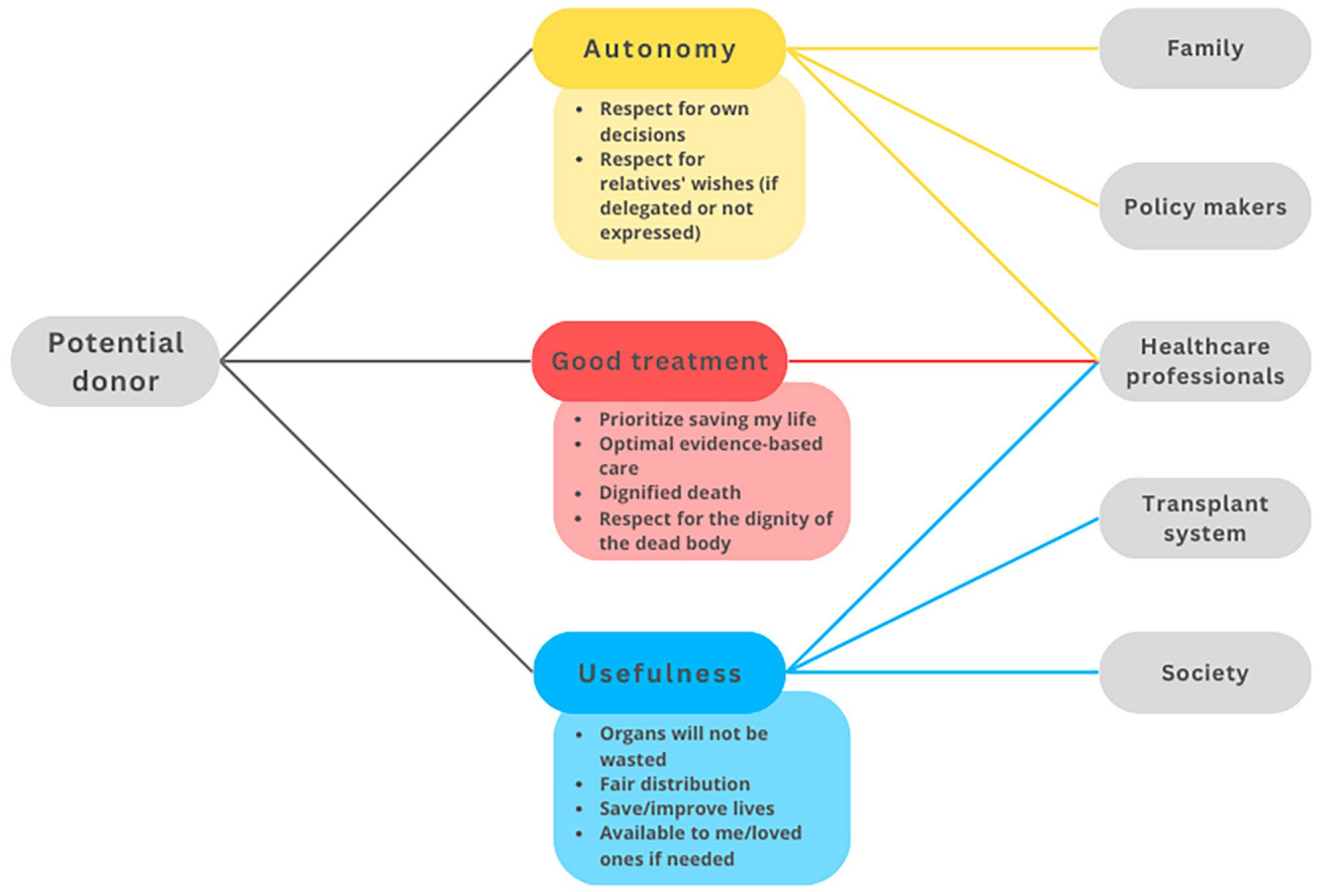
Trust relationships from the potential donor’s perspective. From the perspective of the potential donor, autonomy is linked to respect for the personal decision, but also to respect for the wishes of the family (if the potential donor has delegated his/her decision to the family or has not expressed a decision during his/her lifetime). Thus, autonomy in this context depends on the person (who can make a decision while alive), the families (who should reach an agreement), the policy makers (who establish donation policies) and the HCP (who must respect the wishes of the person and/or his/her relatives). The second object of trust is the expectation that one will receive the best available treatment, in the sense that the HPC will try to save life first, provide optimal evidence-based care and respect the body. Finally, the third object of trust focuses on organ use. The potential donor trusts the HCP because they will perform a removal under the right conditions to ensure the viability of the organ. The potential donor also trusts the transplant system, which is responsible for ensuring that the organ is distributed fairly in order to save lives. Finally, the potential donor must trust society because it is necessary for other people to donate so that there will be organs for them or their loved ones if they need them

**Fig. 3:**
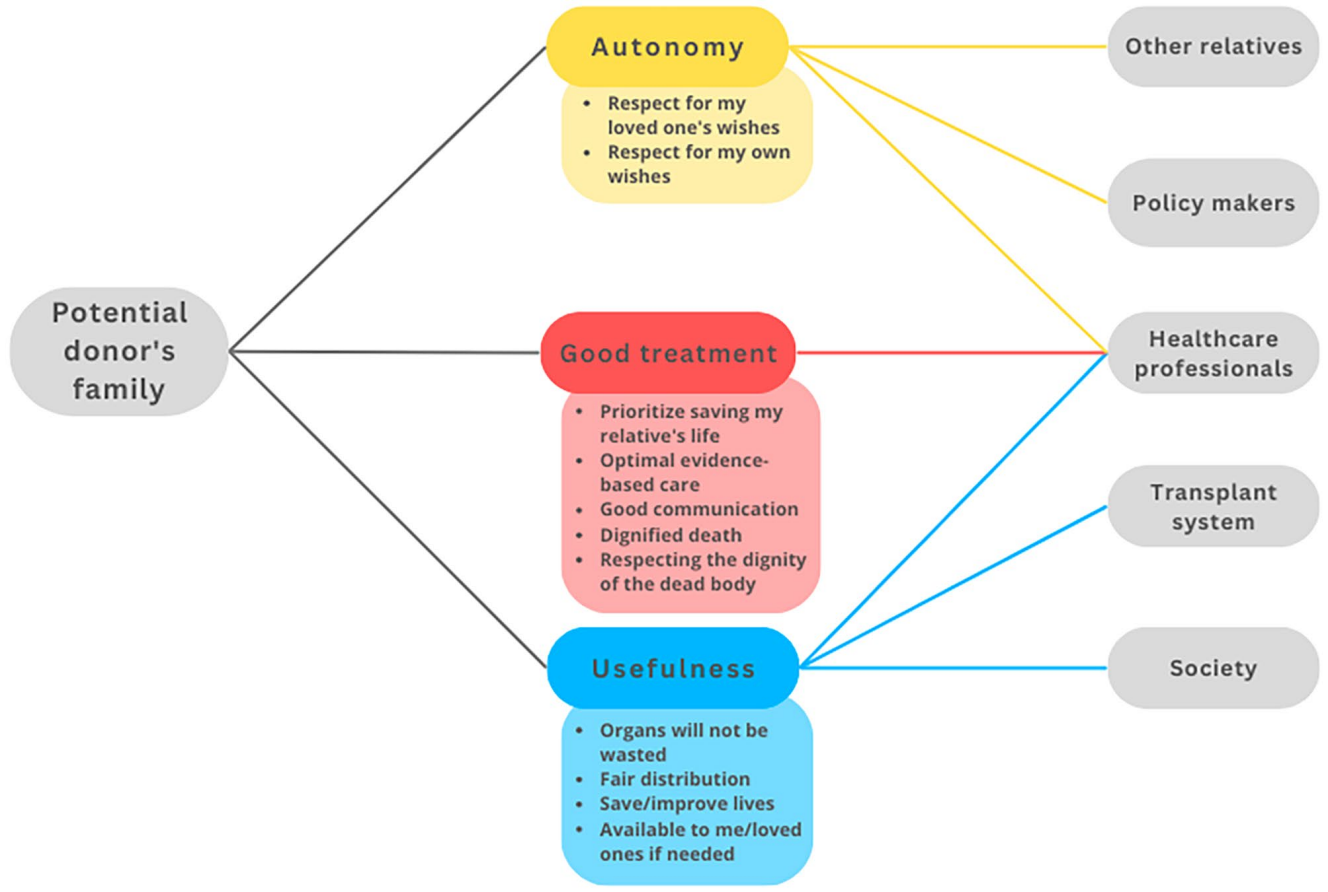
Trust relationships from the potential donor’s family perspective. From a family perspective, autonomy is linked to respect for the wishes of their loved one and/or for their own wishes. Autonomy here refers to the following trustees: the other relatives (there must be agreement among them), the policy makers (who create the donation policies) and the HCP (who must respect the wishes of the person and/or his/her relatives). The second object of trust is the hope that their loved one will not be harmed and will be treated in the best possible way. On the other hand, there is also the expectation that the family will be honoured by maintaining good communication with the agents involved. Finally, for the third object of trust, families hope that their loved one’s organs will not be wasted, that they will be distributed fairly, that they will save lives, and that they too will be able to receive an organ at another time if necessary in the future. For this third object of trust, families trust both the transplantation system and society as a whole

**Fig. 4:**
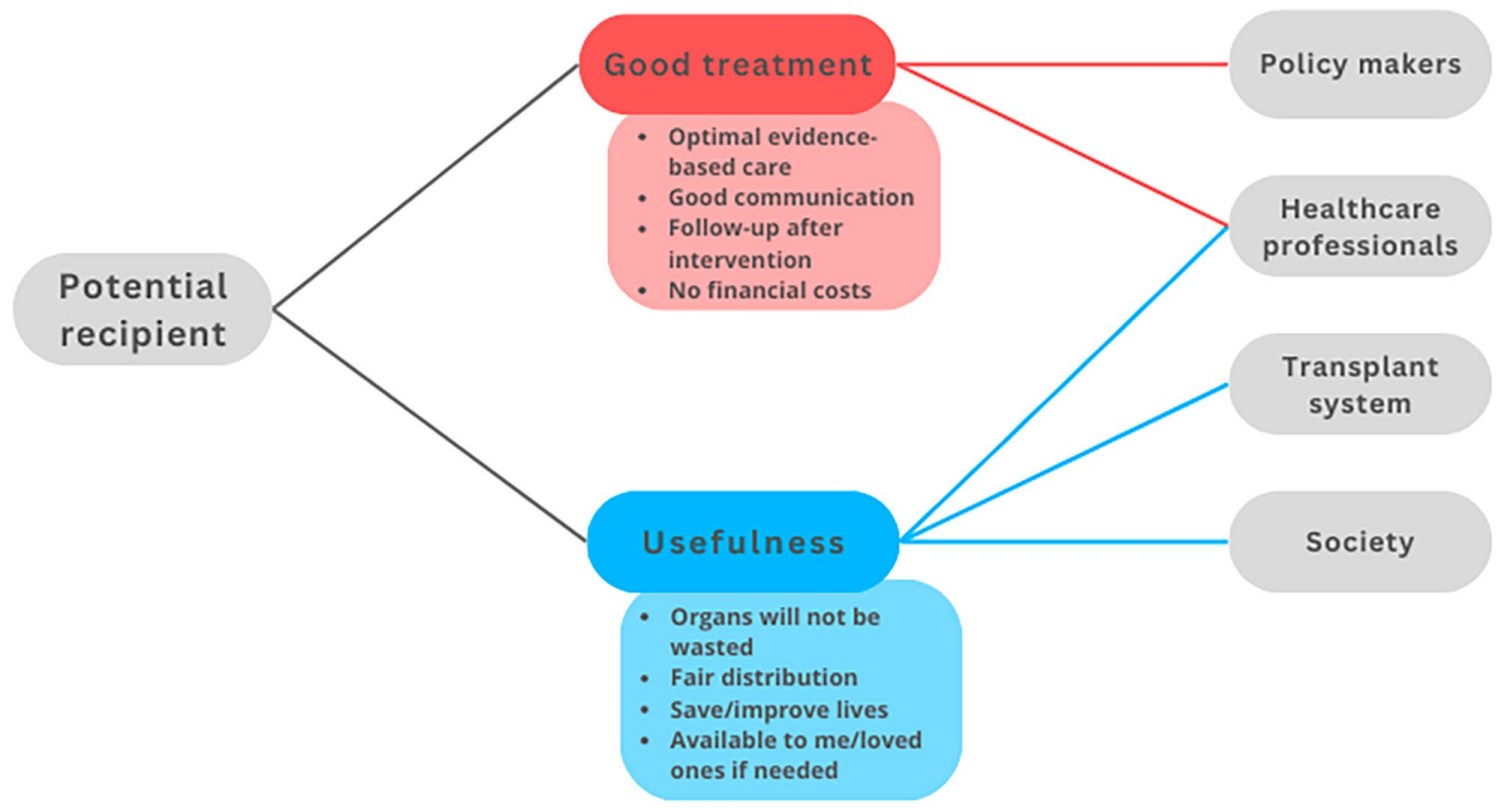
Trust relationships from the recipient’s perspective. From the potential recipients viewpoint, the objects of trust can be grouped alongside various ethical considerations, from ensuring that donor ODT and allocation are organised in an efficient manner to maximise the advantages obtained from the scarce resource of donor organs. Potential recipients also need their doctors and the members of the health care team to assist them in making the right decisions concerning the acceptance of certain types of organs (e.g. living or deceased donor organs, marginal grafts, HLA and blood group matching, domino transplantation, split liver grafts) and the timing of the transplantation. The trust in the health care teams also extends to the period during and after transplantation, as organ transplantation is a major surgical intervention, whose prospects of success depend on the experience of the surgical teams and recipients will need lifelong immunosuppressive therapy and support in maintaining the best possible organ function. Finally, in the case of recipients, it is important that the law does not base a system of organ procurement based on economic cost so that the organs can be purchased at a reasonable cost as this would lead to inequality between people from different social groups. Besides, in registering for a transplantation program, potential recipients generally need to trust that they have at least a slim chance of getting a suitable donor organ and that the surgical intervention will improve their health and/or life expectancy. For the majority of potential recipients, this entails the belief that donor organs are allocated fairly, i.e. that the allocation system satisfies basic principles of distributive justice such as equality, effectiveness, or medical urgency, although people seem to differ on their personal view of what a fair distribution of donor organs should look like [30, 31]

**Fig. 5:**
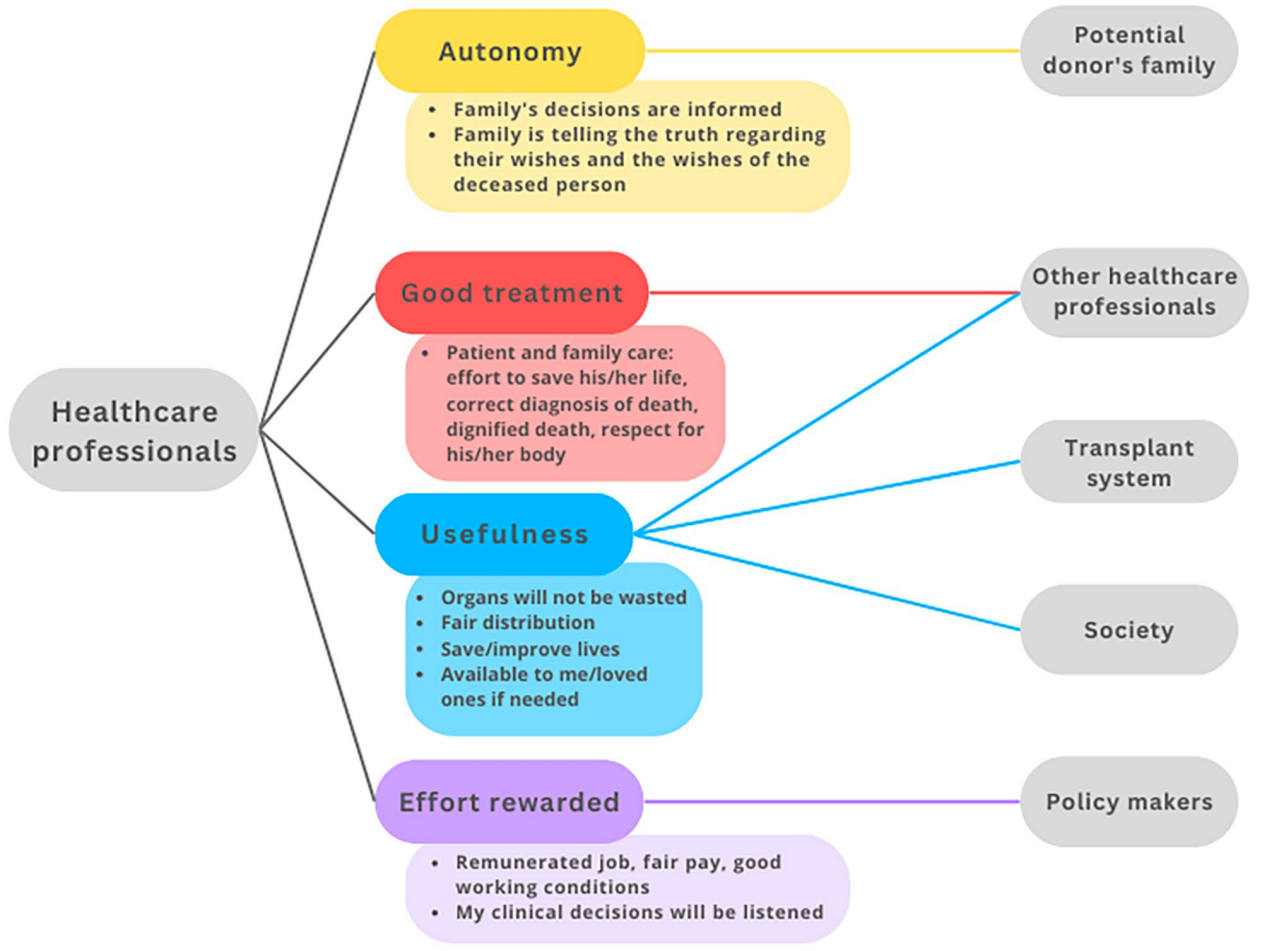
Trust relationships from the health professional’s perspective. Professionals involved in ODT must trust that the patients’ families understand what is being communicated to them (e.g. the diagnosis of death). Also, this comprehended knowledge triggers autonomous and informed decisions (authorisations or refusals). And even more relevant, HCP must believe when relatives act as witnesses that they are telling the truth with respect to their own wishes to donate and with respect to the wishes of the deceased person. On the other hand, HCP professionals must rely on the professionalism of their peers for the whole transplantation process to work. For example, the surgeon who removes the organs must be confident that the diagnosis of brain death has been well established by the scientific community and the professional who diagnosed it in that patient. In another sense, for transplantation to work, HCP must be convinced that it serves a good purpose. This means that the whole transplant system works well and that the organs removed are not wasted, are distributed fairly, and improve or save someone’s life. In addition, HCP must trust that society will continue to donate and organs will be generated for transplantation. Finally, health professionals must be confident that their work is recognised by the policy makers and compensated with a fair financial return for the effort invested, and that labour laws provide decent working conditions

In the following, we aim to construct a model of trust relationships by focusing on relationships between trustors, trustees, and objects of trust that are typical in ODT. We identified four main *trustors*: the potential donor (Fig. 2), the family (Fig. 3), the potential recipient (Fig. 4), and the healthcare professionals (HCP) (Fig. 5). For the sake of clarity, we first disclose the trust relationships that stem from each of these trustors independently. However, it is important to note that these four trustors are part of an interconnected network.

All objects of trust that appear in each trust relation have been selected by considering the existing literature and studies on the perceptions and opinions of agents towards ODT. However, since we aim to model trust distribution, we have grouped these diverse realities into several categories as objects of trust that however need not be considered an exhaustive list and do not imply that all objects of trust work in the same way for all relations. We have thus considered autonomy, usefulness, good treatment and effort rewarded as four potential objects of trust that can potentially well represent these trust relations, although future empirical work could help test the accuracy and exhaustiveness of this selection.

At the agent’s level, a *potential donor* is any living person capable of making a decision within a jurisdiction where either living or deceased organ donation is possible. By *potential donor family* we mean those people close to the deceased person who can influence the decision, either because they are consulted, or to the extent that they can react to, or oppose the retrieval of organs. By *potential recipient* we mainly refer to patients with terminal organ failure who are waiting for an organ transplant, i.e. who are on a waiting list or are considering registration for a transplantation program^3^. By *healthcare professionals* (HCP), we mean those directly involved in the process of identifying potential donors, information, request, procurement, and transplantation of organs. Figures 2, 3, 4 and 5 depict relations between the elements of trust –trustor, elements of trust, trustee– from the perspective of these four actors.

At the institutional level, the *DTS* refers to the complex network of professionals, organisations, and regulations involved in the process of ODT, with the goal to facilitate the successful transplantation of organs and tissues from donors to recipients. Amongst the DTS, *policy makers* are those involved in the creation and promotion of regulatory frames for transplant activities, including transplant laws, clinical protocols, algorithms design, communication strategy, public education, etc. Finally *society* involves all citizens who may become potential donors, families, HCPs or potential recipients at some point in their lives. On occasions, individual actors may even play several roles simultaneously.

All of these relations of trust from the perspective of these four actors can be influenced by different factors, such as the potential donor and recipient’s personal experience with the healthcare system or specific institutions and healthcare teams, the general public level of trust in the healthcare system, as well as the occurrence and public discovery of irregularities or corruption in the allocation system, personal beliefs concerning the effectiveness and meaningfulness of organ transplantation, and the personal conceptions of the bodily integrity and alienation [32, 33].

While this model allows for synchronous analysis of trust relationships, it is essential to acknowledge the dynamic nature of trust, necessitating the inclusion of a temporal dimension. Trust can be either strengthened or undermined over time based on the acquisition of information or personal experiences. For example, the manner in which healthcare professionals handle pre-mortem care can significantly impact the decision of the family to donate, as well as their overall trust in the healthcare providers or the system itself. Moreover, trust and mistrust can spread to others through interpersonal communication, media, or social networks. Positive experiences encountered by recipients or their families can cultivate a sense of solidarity or reciprocity, prompting them to express a willingness to donate [34]. Conversely, the communication of negative experiences can breed distrust among individuals and subsequently influence their inclination to donate. Trust is contingent upon one’s past lived experiences and external information, shaping their perceptions of the healthcare system and donation process.

### The media

The media – both traditional and social media – are key in preserving or debilitating public trust in organ transplantation, as they are the main vehicle and source of massive information to the public. Media and social media can be either supportive of ODT– if they feature favourable news and provide publicity for transplantation medicine – or a threat to that endeavour, as negative news can spread fast and escalate into a scandal [35]. Scandals are something the transplant organisations do care about, as trust is something that might take time to build and be easy to lose. Regular meetings with the media are part of some transplant organisations’ strategy to preserve and promote public trust, where journalists are reminded of the importance of preserving the anonymity of donors and recipients, and the importance of depicting the achievements and breakthroughs of transplantation as those of a system, rather than individuals’ [36, 37]. Even more important is the way scandals are avoided and managed to prevent them from escalating. Handling misinformation, the speed it can spread and the harm it causes is an evolving situation. The agencies trying to manage such things spend increasing time and resources on such matters all in order to stop the spread of mis- or harmful information that can erode trust. In Spain, the National Transplant Organization (ONT) has a proactive policy that involves quick reactions to scandals in the media and providing explanatory information suggesting why the event concerns an isolated case.

### Social contexts and identities

Building and maintaining trust are considered essential components of successful ODT systems in developed countries [38]. In these countries, general healthcare is usually available both via public and private providers. Individuals (often on higher income) may decide which avenue to access services based on a variety of personal and life factors (e.g. urgency, wait times, quality of life, availability of treatments, security, prior experiences, etc.). In terms of the trust, having (more) choices to decide where/when/how to seek healthcare tends to affect the trust dynamic, but not necessarily the levels of trust in either provider [39]. The nuances of trust in and between public and private healthcare systems in relation to ODT are less well known, with comparative global research tending to focus on more tangible outcome measures such as consent and a number of transplants (Global Observatory on Donation and Transplantation). Additionally, people are complicated and as populations continue to become more diverse, learning more about what influences trust at an experiential level helps us better understand where and when individuals are likely to direct their trust within a system [40].Culture, including religion and ethnicity, are crucial elements for the perception of ODT and, hence, how trust in ODT is formed. Briefly, we know that the impact of ethnicity [41], culture, and religion [42] are highly cited factors for individuals supporting deceased organ donation or not. At the same time, people from minority ethnic backgrounds have historically higher levels of mistrust in policymakers and related agents (e.g. the police) which can potentially stoke even higher levels of mistrust when these organisations are seen to overlap with matters of healthcare – e.g. policy and law making [43]. Level of education is a known factor in people’s awareness of ODT and by extension, their trust in the system as ‘not knowing enough about it’ are still top reasons why people have opted out of (or feel uncertain about) ODT. On the other hand, some evidence suggests being female, younger and lower-income are all positively associated with people who want to become organ donors [44]. To what extent all these demographics are a result of people’s social roles (e.g. wife, carer, mother), their social class and status as well as individual circumstances and life events are not very well understood. Understanding how these highly personal factors interrelate and overlap is vital to conceptualising and better understanding how trust is built and sustained in complex systems.

## Trust map

**Fig. 6:**
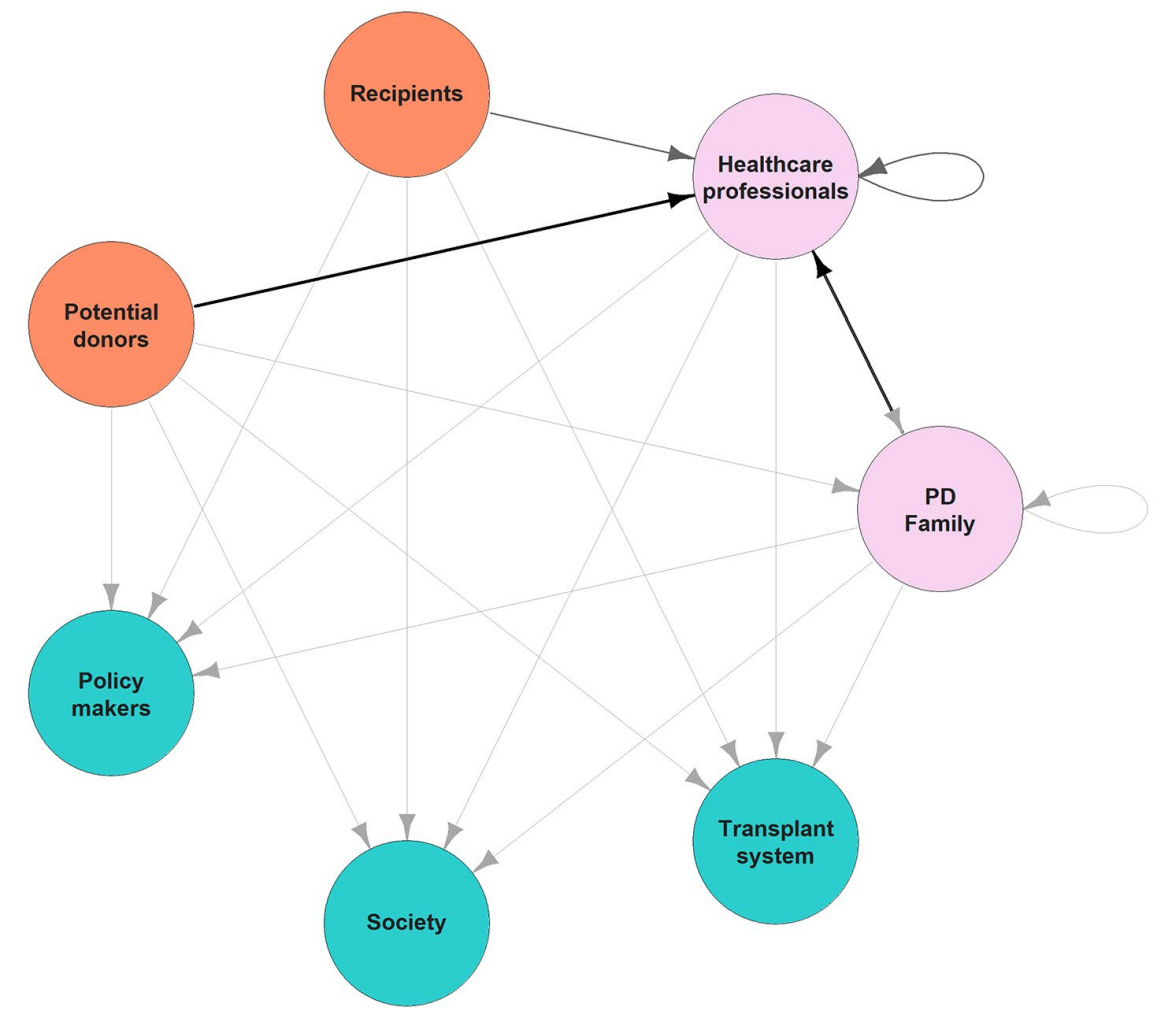
Proposed trust distribution network. Colour and width of edges represent the weight of the links. Directions are represented with arrows

In Fig. 6, the net graph^4^ aims to represent how each of the trustors and trustees involved deposits or receives different objects of trust. We have developed this map by operationalizing the previous trust distribution figures for each of the trustors and combining them into a single trust network. This has been done by assigning a number for each of the possible relations between agents in both directions. A relation’s value is considered to be null if an agent does not transfer any objects of trust to another agent. But, if it does deposit some, the relation’s value increases by one unit for each such object. The resulting network is directed and gradient, as seen in the figure.

Note that some of the trustors deposit several objects of trust in the same trustee. This reality is displayed in the graph as having a wider edge. For instance, recipients trust the government to provide them with good treatment, but also to foster solidarity (which will eventually get them an organ). This can be seen in the graph with a bolder edge between recipients and the government than between recipients and other trustees. It is also worth noting that the graph has two loops (links with the same trustor and trustee), which represent specific network linkages. Practitioners show trust in the professionalism of their colleagues. Families rely on other relatives to respect their autonomy. We believe that this is better represented as a loop rather than a distinct connection because practitioners and family members are both depositing and receiving trust from their peers.

We do not intend to run additional network analyses or calculate centrality measures because this is a preliminary concept. Empirical research can potentially use this network to assess how strong the ties are, how thick the network is, or the existence of new relationships that have not yet been considered. We believe that this model offers a good prospect and that it can be an interesting and promising path to explore in future empirical studies.

Thus, according to the graph, we can see that the three blue nodes are pure trustees (government, transplant system, and society), having all arrows directed to them and none directed towards others. On the opposite side, the two orange nodes are pure trustors (recipients and donors), having only arrows oriented toward other nodes but no arrows toward themselves. Lastly, the two violet nodes are hybrid (family and practitioners), having arrows directed toward both themselves and others.

**Fig. 7:**
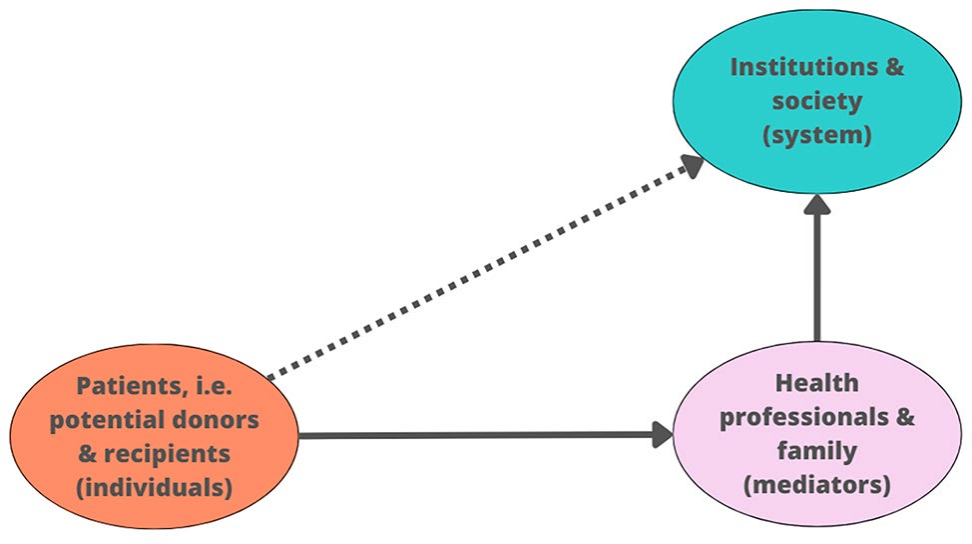
Three-level trust map

This points in the direction of a more general interpretation of each of these three groups, as represented in Fig. 7: there are patients (recipients and donors), who need others (family and practitioners) to mediate with the macro-level that runs the system (government, transplant system, and society). Therefore, each of the nodes has different roles in the global network, and this also means that factors affecting trust will have differential impacts on trust depending on the level they affect.

### Examples of factors affecting trust map

The graph we presented above is an ideal and static one. Real social networks are usually dynamic and change their connections when influenced by different factors. As much as the number of influencing factors is virtually unlimited, in this section we aspire to offer some examples showing the way we expect factors to shape trust relationships.

Let us consider an actual event that happened in Germany in the decade of the 2010s. By the beginning of 2010, the trend for deceased organ donors started to decrease. In 2012, there was a scandal in the media accusing some doctors of falsifying documents in order to get better positions for their patients in waiting lists [47]. Although the organ donation rates were already declining before the scandal became public, some studies suggest that this negative trend in organ donation rates was reinforced during the years after the scandal, even though the global trend for other European countries was the exact opposite, having growing numbers of donations [48].

**Fig. 8:**
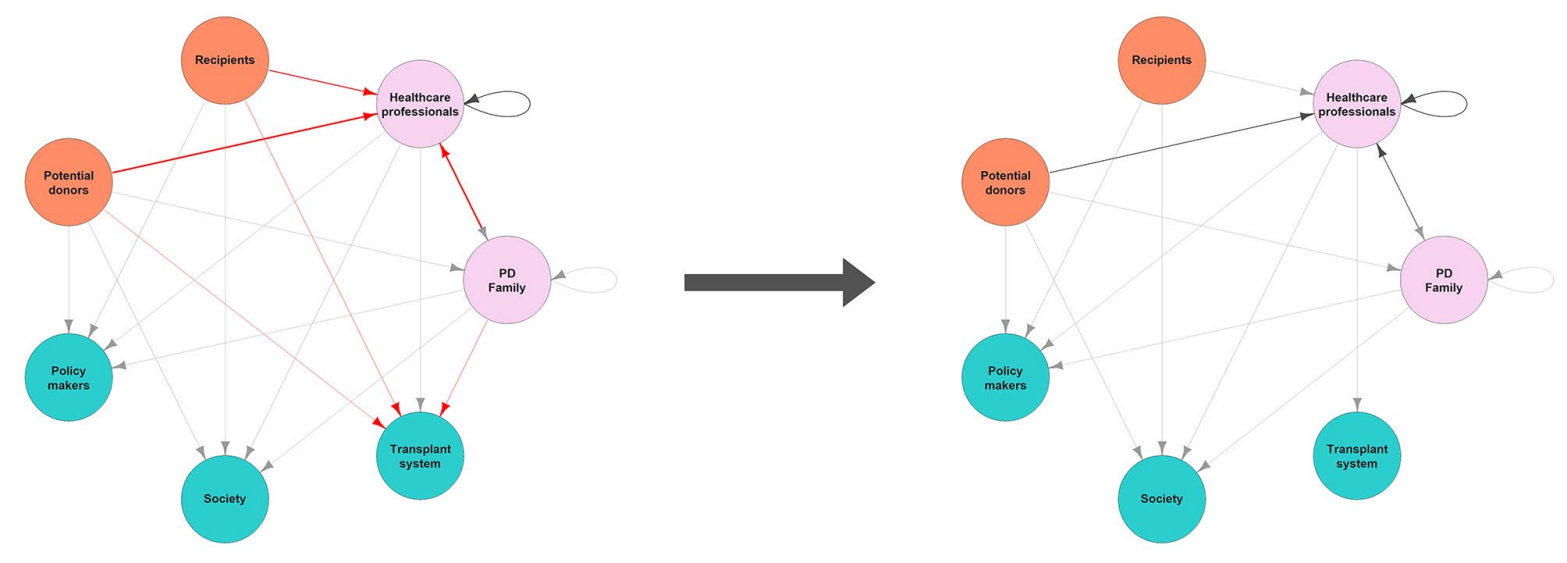
Trust network before and after the effect of news about corruption in organ allocation. Trust arrows from donors, recipients, and families addressing the transplant system fully disappeared. Trust arrows from donors and family addressing health professionals reduced their width as they still have some unaffected objects of trust on them (i.e. good care)

A plausible explanation for this effect is that the information spread by the media affected people’s willingness to trust health professionals to fulfil one of the objects of trust: the usefulness and fairness of the organ distribution. If a potential donor cannot trust that the organ transplantation system is fair, it is plausible that they will not be willing to participate in it. But this event has a limited range of effects. The potential donor is no longer trusting (or at least not in the same degree) health professionals to fairly allocate their organs, but this needn’t affect the trust that they still have in their families to respect their decision, or even the trust in the same health professionals to cause them no harm. In line with our proposed model, news about corruption partially affect the trust map distribution, as shown in Fig. 8. In this figure, we propose that news about corruption in organ allocation are negatively affecting some of the trust relationships: potential donors and their families will no longer trust either health professionals or the transplant system to fairly allocate the organs (object of trust: usefulness/fairness), and recipients will analogously lose their trust in the transplant system and HCP to be treated fairly (object of trust: usefulness/fairness).

## Discussion

This study explored trust relationships from the perspective of the main actors involved in ODT. As expected, the findings are complex. We have developed a series of Figs. (2–5) to clarify who are the main trustor and the trustees, and what are the main objects of trust in each relationship. The Figs. (2–5) could also be interpreted from the perspective of tasks that different trustees are supposed to fulfil: to respect autonomy, good treatment, etc. The trustees are the persons supposed to fulfil or help to fulfil these tasks (or even obligations). The model shows that the government, society and the transplant system are mainly to be addressed as trustees in empirical research, not as trustors. We believe that thisconceptual model can assist ODT institutions and policymakers in determining which elements (objects of trust) to focus on if they want to increase the trust of the various actors in the ODT process or system. By reading Figs. 2, 3, 4 and 5 from right to left, transplant system workers, HCP, and policymakers can visualise which are the primary objects of trust to which they should pay attention in order to increase trust in each of the trustors. In addition, there are some tensions or nuances that might be clarified.

### Autonomy

The ethical justification for organ procurement, both living and cadaveric, is generally considered to rest on the principle of autonomy, i.e., the donor’s own consent^5^ and/or the authorisation of family members [4]. On the one hand, potential donors need to trust that their relatives will respect their wishes after death [49]. On the other hand, relatives who authorise organ removal will do so if they perceive the professionals, the transplantation system, and the healthcare system to be trustworthy [28]. The involvement of families is usually based on the belief that they know what is best for the potential donor, because they know the person better than the medical team, and will therefore act as guarantors of the deceased’s wishes^6^ [50]. Organ removal against the will of the family can not only damage the doctor-patient-family relationship [51], but the family may also lose trust in the transplantation system and the healthcare system. Giving the final decision to the families, on the other hand, is equivalent to devaluing the deceased’s decisions and contradicts the respect for individual freedom [52–55].

Some tensions, such as the one between the deceased’s autonomy and the autonomy of the family, cannot be solved with a conceptual model but with an empirical study. On the one hand, individual autonomy, meaning the freedom to live one’s own life according to one’s own preferences, without excessive interference from others [56, 57], may conflict with family-related autonomy. On the other hand, sometimes the family functions as an autonomous social entity, with the final authority to make clinical decisions in ODT. The way how society (even in the same society this varies depending on the person) or people prefer to respect the family vs. individual autonomy can be related to some cultural and healthcare aspects [58]. What might erode trust, respecting the deceased’s desires over the family or vice versa? We cannot answer this question, however, we can point out that trust can rise or decrease based on cultural background if family autonomy is valued over the deceased’s one. All of these factors may have an impact on trust.

### Information and transparency

Another ethical issue is the relationship between trust and transparency, which is not clear: the more transparent a system is, the more reliable it becomes. The interconnection between transparency and public trust cannot be taken for granted but must be demonstrated, as increased public knowledge of morally challenging practices and regulations may increase doubt and public concern. Contrary, less public awareness of transplantation regulation and actual practice can contribute to higher levels of public trust. In a representative survey, carried out in Spain [10], the majority of the population surveyed stated that they trust the public health system, especially the donation and transplant system, considering it as transparent. However, the majority ignored that Spain is governed by an opt-out system or they recognised that they are unaware of the consent model. In addition, more than half of those surveyed felt uninformed in some way about the necessary requirements to be an organ donor in Spain.

Transparency is considered essential for building and maintaining public trust in ODT. For the people tasked with creating a transparent system, providing information freely and accessibly is considered fundamental. However, in general to date, there has been little focus on the people on the receiving end of such information and how that information is made meaningful and useful to them.

As we have demonstrated that trust relationships in ODT are complex and rely on the interplay of multiple actors and highly personal factors. Learning more about how information is translated into meaning by the consumers, will create new contexts to evaluate the quality of the information provided.

These are important considerations and areas for future research, as the science of ODTcontinues to evolve at pace, we need to develop more holistic approaches to transparency that focus on the quality of information, and how it is meaningful *and* useful to the various consumers.

### Reciprocity and solidarity

Cadaveric organ donation is a unique form of exchange. It can be based (a) on the selfless act of the deceased or their loved ones to donate organs for transplantation without any expectation of direct reciprocity (altruism), or (b) on the fact that in a certain sense people expect the system to be reciprocal, on the believe that one day the person might have an organ if it is need it (reciprocity). In any of these two situations, people may still have direct interests that they wish to see respected, such as their autonomy, dignity, and the appropriate care before and after death. Consequently, the willingness of individuals and their families to donate is linked to their trust that their interests will be taken into account. Moreover, they want to ensure that their organs will not be wasted and will be used to help others. This wish may be based on the comforting idea that helping others gives meaning to death, or on a sense of duty or moral obligation to others that is required of every member of society, or on the idea that there is indeed reciprocity in that we as a society benefit from having an effective ODT system. Therefore, although organ donation is usually seen as the epitome of altruism, it may also (or instead) be based on solidarity [4].

### The body concept

Transplantation medicine creates its own concepts of the availability of bodies that do not correspond to everyday practice (e.g. cultural death rituals) [6, 59, 60]. The body seems to be modifiable by means of medical techniques, but not all boundaries between body and technology, life and death, and own and foreign disappear completely [6]. This grey area between life and death represents a taboo zone for many laypersons vis-à-vis the medical system, related to questions about prolonging the dying process or dealing with the dead (resting with the dead). If this taboo is not just addressed openly, it creates uncertainty. This uncertainty has an impact on trust towards the medical staff and also towards the system. Besides, an important part of building trust in OD involves treating the donor with dignity. Dignified means a special situation. The dying process is artificially interrupted. There is a situation of “to speak ill of the dead” [6]. This survey showed that these points were in Transplantation medicine a kind of taboo but on the other hand real fears of lay persons. These aspects are called boundary shifts that trigger uncertainty [6]. For us, it is the task of transplantation medicine to ensure trust at these shifts in boundaries. And the third, is the objects related to the purpose of the donation. To each of these objects correspond agents in whom each of the objects of trust is placed. They are the agents on whom it depends whether this trust is fulfilled or not.

### Scope and limitations of the conceptual model

In this paper, we have developed a conceptual model of trust that can be applicable for post-mortem donation and transplantation. Thus, living donations might have different trust relationships. Although living donation is not covered within the model provided here, we hope our work can stimulate future research about trust relationships in living donation.

One of our model’s limitations is the notion that personal ideas, values, and beliefs are influencing variables in trust relationships. We believe that some traits, such as ethnicity, religion, or personal values among potencially many others, may influence how the trust relationship is envisioned and formed. As a result, considering these aspects “just” as influencing factors, as we have, can be problematic. In our approach, however, influencing variables are critical and constitutive of the concept of trust.

Besides, in this article, we focus on the concept of trust among social actors, such as individuals, groups, institutions, and systems like the healthcare system. However, there are other forms of trust that exist, including the trust between organ recipients and their own bodies. Humans are socialised from an early age to rely on their body’s immune response for survival, such as when experiencing a fever. When this response is suppressed, organ recipients may struggle to trust their own bodies. To regain trust, they must establish a new normality through medical measurements and a trusting relationship with their doctor. Additionally, they must adapt to a new way of life and develop their own scales to interpret their state of health, based on their pre-transplant state and their performance in daily activities after the transplant [60].

Finally, this paper presents an initial model, but it needs to be discussed with the different stakeholders to gain insight into their perspectives. Further research is therefore needed to test whether the model works from the perspective of the actors involved, in particular coordinators, doctors, nurses, patients and their families, before it can be used for empirical research.

## Conclusions

We propose a conceptual model to understand and analyse the role of trust and its dimension and relationships in ODT. We have sought to clarify what trust is in general, in the healthcare system, and in the ODT system in particular. We hope thisconceptual model may serve as a foundation and guide for future empirical research, such as the development of scales or questionnaires to address trust in regard to ODT from the perspective of the different actors involved in the process. Future work could consider empirical studies in the population to measure: objects of trust, trustees, etc. Moreover, these studies should be cognisant of the roles of trustees and trustors – for example - “government”, “transplant system” and “society” are not an agent, they are not an entity. This is reflected in the map because they do not “place” trust in anyone.

This conceptual model can inform stakeholders and policymakers seeking novel measures to study trust to enhance the effectiveness of transplantation medicine.

## Data Availability

Data is included in the manuscript.

## Footnotes

1 We have taken the different consent models into consideration to develop this conceptual model, and, on this basis, our model of trust relationships in ODT is expected to be applicable for both opt-in and opt-out. Empirical applications would be required to explore whether and how issues of trust differ in both systems.

2 We use the terms mistrust and distrust indistinctly.

3 One could also argue that all people living in a country with an active transplantation program can be regarded as potential recipients as they may all someday suffer terminal organ failure and be confronted with the decision to enrol or not to enrol in a transplantation program or even to decide to accept or not to accept certain types of organs (e.g. organs from living donations, marginal grafts i.e. grafts of minor quality or from elderly people, domino transplantation, split liver grafts). However, we restrict our considerations to patients that are currently awaiting or considering organ transplantation as a surgical intervention for their organ failure, as they are directly dependent on the distribution of and access to donor organs and, hence, directly confronted with the issue of trust in the transplantation system.

4 The graph was created by using the igraph package for R [45, 46].

5 Individuals can express their consent in different ways. Jurisdictions with an opt-in system presume that people who fail to register their consent actually refuse organ donation, whereas countries with opt-out presume that individuals who fail to refuse organ donation actually consent. Only if individuals were informed that their silence will be interpreted as an implicit consent (in opt-out) or refusal (in opt-in), then failure to express a preference over organ retrieval could be taken as a valid expression of autonomy. In practice, many individuals have mistaken beliefs about the consent system that rules in their jurisdiction. Therefore, respect of individual autonomy is problematic in any presumptive policy, and this is particularly true in opt-out countries, where citizens are overall less aware of their consent system than in opt-in countries [5, 15]. Whether or not these differences impact trust relationships should be explored empirically.

6 Families are usually consulted, in both opt-in and opt-out countries, when the deceased have not expressed any preferences with regard to organ donation. In this case, in the absence of any written record, relatives are considered to be in a better position than health professionals to make a decision that is respectful of the deceased’s wishes, if any. That being said, the family is often consulted even when there is a written record of the deceased’s wishes. Indeed, the deceased may have changed their mind between the time they recorded their wish to donate and the time of death.
