# Correction to: Mapping trust relationships in organ donation and transplantation: a conceptual model

**DOI:** 10.1186/s12910-024-01026-y

**Published:** 2024-03-05

**Authors:** María Victoria Martínez-López, Leah McLaughlin, Alberto Molina-Pérez, Krzysztof Pabisiak, Nadia Primc, Gurch Randhawa, David Rodríguez-Arias, Jorge Suárez, Sabine Wöhlke, Janet Delgado

**Affiliations:** 1 Department of Philosophy I, FiloLab-UGR, University of Granada, Granada, Spain; 2 Ethical Legal and Psychosocial Aspects of Organ Transplantation (ELPAT), European Society for Organ Transplantation (ESOT), Padua, Italy; 3 School of Medical and Health Sciences, Bangor University, Bangor, UK; 4 Instituto de Estudios Sociales Avanzados (IESA), CSIC, Córdoba, Spain; 5 Dept Nephrology Transplantation and Internal Medicine, Pomeranian Medical University, Szczecin, Poland; 6 Institute of History and Ethics of Medicine, Medical Department, Heidelberg University, Heidelberg, Germany; 7 Institute for Health Research, University of Bedfordshire, Bedfordshire, UK; 8 Department Health Sciences, Faculty Life Science, Hamburg University of Applied Sciences, Hamburg, Germany

Correction to: BMC Medical Ethics (2023) 24:1

10.1186/s12910-023-00965-2

Following publication of the original article [1], there are few text that has been changed in the acknowledgement section. So, the section should read from “The authors thank Maite Cruz Piqueras, Anja Marie Bornø Jensen, Eva Martín Nieto, Jorge Ruiz Ruiz and Silke Schicktanz for their contribution. MVML also thanks the philosophy doctoral students of the University of Granada for their contribution. This research has been conducted as part of the following projects: “Research in Ethics of Organ Donation and Transplantation” (INEDyTO) [MINECO FFI201 88913-P]; “Epistemological and bioethical analysis of the criteria for determining death” (ABCDm) MICINN: PID2020-119717GA-I00]; “Moral science and institutional design lab” (MSIDLab) [PID2020-119791RAI00].” to “The authors thank Maite Cruz Piqueras, Anja Marie Bornø Jensen, Eva Martín Nieto, Jorge Ruiz Ruiz and Silke Schicktanz for their contribution. MVML also thanks the philosophy doctoral students of the University of Granada for their contribution. This research has been conducted as part of projects: INEDyTO 2 (PID2020-118729RB-I00), ABCDm (PID2020-119717GA-I00), and MSIDLab (PID2020-119791RAI00) funded by MCIN/AEI 10.13039/501100011033.”

Also, there is change in the funding section. So, the text will read from “Research in Ethics of Organ Donation and Transplantation” (INEDyTO) [MINECO FFI201 88913-P]. Open Access funding provided thanks to the CRUE-CSIC agreement with Springer Nature.” to “Research in Ethics of Organ Donation and Transplantation” (INEDyTO) [MINECO FFI201 88913-P]. Grant PID2020-118729RB-I00 funded by MCIN/AEI 10.13039/501100011033. Open Access funding provided thanks to the CRUE-CSIC agreement with Springer Nature.”

The original article has been corrected.
